# Supplementary material for: Disruption of Human Papillomavirus 16 E6/E7 Genes Using All-in-One Adenovirus Vectors Expressing Eight Double-Nicking Guide RNAs
Source: Int J Mol Sci. 2025 Sep 5;26(17):8685. doi: 10.3390/ijms26178685 (PMC12429375; doi:10.3390/ijms26178685)
Supplement: Supplementary file 1 [file ijms-26-08685-s001.zip › ijms-3837478-Supplementary Information (Sup Materials and Methods )R2.pdf]

Supplementary information

Supplementary Materials and Methods

plasmids

The construction of the destination vectors pDEST-CLXSN and pDEST-CMSCVpuro has been previously described [60]. The vector pDEST-CMSCVbsd was similarly constructed from pCMSCVbsd. To create pCMSCVbsd and pCMSCVzeo, the HindIII-BanIII puro segment of pCMSCVpuro was replaced with a HindIII-BanIII bsd segment and a HindIII-BanIII zeo segment, respectively, which were originally derived from pEF6-MycHis-A (Invitrogen) by PCR using primers 5'-GGAAGCTTCAGCACGTGTTGACAATTAATC-3' and 5'-CATCGATTAGCCCTCCCACACATAACCAGAG-3', and pDONR/zeo (Invitrogen) by PCR with primers 5'-GGAAGCTTCAGCACGTGTTGACAATTAATC-3' and 5'-CATCGATTAGTCCTGCTCCTCGGCCACGAAGT-3', respectively. The HPV18 E6E7, E7, and splicing donor mutant E6SD segments were cloned into destination vectors to generate pCLXSN-18E6E7, pLXSN-18E7, pCLXSN-18E6SD, and pCMSCVbsd-18E6SD.

The construction of the destination vector pDEST-CL-SI-MSCVpuro has been previously reported [61]. Similarly, pDEST-CL-SI-MSCVbsd was constructed from pDEST-CMSCVbsd. The gRNA-GFP-T2 plasmid, which was a gift from George Church (Addgene plasmid # 41820) [3], was amplified by PCR using primers 5'-GTTTTAGAGCTAGAAATAGCAAG-3' and 5'-CgggaaagaGTGGTCTCATcaagatatataaagccaaga-3' and was re-circularized by in-fusion reaction using the In-fusion HD kit (TAKARA) to modify the 3'-end of the U6 promoter, generating pCR-Blunt2-U6/H1-gRNA_GFP-T2. The target sequence in pCR-Blunt2-U6/H1-gRNA_GFP-T2 was then mutagenized by PCR with primers 5'-CTTTTATACTAACCGGTTTgttttagagctagaaatagca-3' and 5'-AAACCGGTTAGTATAAAAGcggtgtttcgtcctttccaa-3', and was re-circularized by in-fusion reaction using the In-fusion HD kit (TAKARA) creating pCR-Blunt2-U6/H1-gRNA_HPV16AS57G.

The gRNA expression cassette was amplified by PCR using 5'-AAAAAGCAGGCTaagcttAAAAAAGCACCGACTCGGTGCCA-3' and 5'-AGAAAGCTGGGTgaattcAAGGTCGGGCAGGAAGAGG-3', followed by 5'-GGGGACAAGTTTGTACAAAAAAGCAGGCT-3' and 5'-GGGGACCACTTTGTACAAGAAAGCTGGGT-3', and the PCR products were recombined with pDONR221 via BP reaction (Invitrogen), generating pENTR221-U6/H1R-16AS57G. The construct pENTR221-U6/H1R-16AS57G was then recombined with pDEST-CL-SI-MSCVpuro

and pDEST-CL-SI-MSCVbsd through LR reactions (Invitrogen) to generate pSI-CMSCVpuro-U6H1R-16AS57G and pSI-CMSCVbsd-U6H1R-16AS57G, respectively.

To generate pSI-CMSCVpuro-U6H1R-GFP-T2, The target sequence in pSI-CMSCVbsd-U6H1R-16AS57G was mutagenized by PCR using primers 5'-GAGCGCACCATCTTCTTCAgttttagagctagaaatagca-3' and 5'-TGAAGAAGATGGTGCGCTCcgggaaagagtggtctcat-3', and circularized using an In-fusion HD cloning kit (TAKARA Bio. Co., Kyoto, Japan). This sequence was replaced in both pSI-CMSCVpuro-U6H1R-GFP-T2 and pSI-CMSCVbsd-U6H1R-16AS57G by PCR with specific primers (Supplementary Table S5) and circularized using an In-fusion HD cloning kit (TAKARA), generating a series of retrovirus vectors such as pSI-CMSCVpuro-U6H1R-16S563g and pSI-CMSCVbsd-U6H1R-16AS507G (Supplementary Table S5).

The promoter-less lentivirus vector, CSII-RfA, was generated from CSII-EF-RfA, kindly provided by Dr. Miyoshi (RIKEN BRC), by AgeI digestion to remove the elongation factor 1α promoter, followed by self-ligation. Entry vector plasmids, pENTR221-(L1-L4)-U6H1R-gRNA-GFP-T1 and pENTR221-(R4r-R3r)-U6/H1R-gRNA-GFP-T2, which had the same sequences as pENTR221-U6H1R-16AS57G except for att sequences and target sequence, were similarly generated from gRNA_GFP-T2 (Addgene 41820).

The target sequences of pENTR221-(L1-L4)-U6H1R-gRNA-GFP-T1 and pENTR221-(R4r-R3r)-U6/H1R-gRNA-GFP-T2 were mutagenized by PCR using sets of primers and re-circularized by in-fusion reactions to create various gRNA expression plasmids (Supplementary Table S7). Sequences of all gRNA expression cassettes were confirmed by Sanger sequencing.

Drug-resistant gene expression cassettes were amplified by PCR from pMSCVneo, pMSCVpuro, pMSCVhyg (TAKARA), and pEF6-MYC-His-A (ThermoFisher Scientific) with primers containing attB3 and attB2 sequences (Supplementary Table S7). PCR products were recombined with pDONR221 P3-P2 to generate five drug-resistant expression plasmids: pENTR221(L3-L2)-PGK-Neo, -PGK-Puro, -PGK-Hyg, and -PGK-Zeo. Three entry vectors, one of each type (pENTR221(L1-L4)-gRNA, pENTR221(R4r-R3r)-gRNA, and pENTR221(L3-L2)-drug resistance), were recombined with CSII-RfA via LR reaction according to the manufacturer's instructions (ThermoFisher Scientific) to produce lentivirus vectors expressing paired double-nicking gRNAs and a drug resistance gene (Supplementary Table S6, Supplementary Table S7).

References:

48. Mali, P.; Yang, L.; Esvelt, K.M.; Aach, J.; Guell, M.; DiCarlo, J.E.; Norville, J.E.; Church, G.M. RNA-guided human genome engineering via Cas9. *Science* **2013**, *339*, 823–826.

60. Kyo, S.; Nakamura, M.; Kiyono, T.; Maida, Y.; Kanaya, T.; Tanaka, M.; Yatabe, N.; Inoue, M. Successful immortalization of endometrial glandular cells with normal structural and functional characteristics. *Am. J. Pathol.* **2003**, *163*, 2259–2269.
61. Haga, K.; Ohno, S.-i.; Yugawa, T.; Narisawa-Saito, M.; Fujita, M.; Sakamato, M.; Galloway, D.A.; Kiyono, T. Efficient immortalization of primary human cells by p16INK4a-specific short hairpin RNA or Bmi-1, combined with the introduction of hTERT. *Cancer Sci.* **2007**, *98*, 147–154.
