# Supplementary material for: Disruption of Human Papillomavirus 16 E6/E7 Genes Using All-in-One Adenovirus Vectors Expressing Eight Double-Nicking Guide RNAs
Source: Int J Mol Sci. 2025 Sep 5;26(17):8685. doi: 10.3390/ijms26178685 (PMC12429375; doi:10.3390/ijms26178685)
Supplement: Supplementary file 1 [file ijms-26-08685-s001.zip › ijms-3837478-Supplementary Table S1-8 revised.pdf]

Supplementary Table S1. List of the candidate DN-gRNA pairs targeting HPV16 genome and the length of 5'-overhang

Name of gRNA				16AS7697g+	16AS57G	16AS63g+	16AS434g+	16AS443g+	16AS507G	16AS616g+	16AS706g+	16AS716g+	16AS717g+	16AS810g+	16AS840g+	16AS841g+	16AS842g+	16AS859G	16AS869G	16AS880g+	16AS890g+	16AS1320g+	16AS1336g+	16AS1438g+	16AS1543G
		nicking site		7697	57	63	434	443	507	616	706	716	717	810	840	841	842	859	869	880	890	1320	1336	1438	1543
			SNVs				A5D3	A5				D2D3	D2D3		A4	A4	A4								
16S7746g+	gCTAATTGCATATTTGGCATA	7746	D3	49																					
16S107g+	gAAAGAGAACTGCAATGTTTC	107	C		50	44																			
16S116g+	gTGCAATGTTTCAGGACCCAC	116	C		59	53																			
16S484g+	gAGCAAAGATTCCATAATATA	484					50	41																	
16S485G	GCAAAGATTCCATAATATAA	485					51	42																	
16S486G+	GCAAAGATTCCATAATATAAG	486					52	43																	
16S490G	GATTCCATAATATAAGGGGT	490					56	47																	
16S493g+	gTCCATAATATAAGGGGTCGG	493					59	50																	
16S498g+	gAATATAAGGGGTCGGTGGAC	498						55																	
16S563g+	gAACCCAGCTGTAATCATGCA	563							56																
16S674G	GAGGAGGAGGATGAAATAGA	674	A3							58															
16S753G	GCAAGTGTGACTCTACGCTT	753									47	37	36												
16S862g+	gTCAGAAACCATAATCTACCA	862	A4											52											
16S875g+	gTCTACCATGGCTGATCCTGC	875													35	34	33								
16S884G	GCTGATCCTGCAGGTACCAA	884													44	43	42								
16S885G+	GCTGATCCTGCAGGTACCAAT	885													45	44	43								
16S886g+	gTGATCCTGCAGGTACCAATG	886													46	45	44								
16S892g+	gTGCAGGTACCAATGGGGAAG	892													52	51	50	33							
16S893G	GCAGGTACCAATGGGGAAGA	893													53	52	51	34							
16S898G+	GTACCAATGGGGAAGAGGGTA	898													58	57	56	39							
16S899g+	gACCAATGGGGAAGAGGGTAC	899													59	58	57	40	30						
16S908G	GAAGAGGGTACGGGATGTAA	908																49	39						
16S912G+	GAGGGTACGGGATGTAATGGA	912																53	43	32					
16S947G	GCTGTAGTGGAAAAAAAAAC	947																			57				
16S948G+	GCTGTAGTGGAAAAAAAAACA	948																			58				
16S949g+	gTGTAGTGGAAAAAAAAACAG	949																			59				
16S1352g+	gAGTCAGTATAGTGGTGGAAG	1352																				32			
16S1353G	GTCAGTATAGTGGTGGAAGT	1353																				33			
16S1354G+	GTCAGTATAGTGGTGGAAGTG	1354																				34			
16S1355g+	gCAGTATAGTGGTGGAAGTGG	1355																				35			
16S1358G+	GTATAGTGGTGGAAGTGGGGG	1358																				38			
16S1394g+	gAGTAGTGGAAGTGGGGGAGA	1394																					58		
16S1471g+	gACTAAAAACTAGTAATGCAA	1471	C																					33	
16S1583G	GATTGGTGTATTGCTGCATT	1583																							40

Supplementary Table S2. List of putative off-target sites selected by CRISPRdirect and the primers to amplify them

gRNA			off-target site							
gRNA	target sequence	Amplicon	Chr(hg19)	Start	End	PCR product size (bp)	Primer site 1	putative off-target sequence	Primer site 2	Target (+)鎖
AS443g+	gAGATGTCTTTGCTTTTCTTC	$1	13	100891192	100891384	193	TCATAGAGTTCTCTCTTAATTCTTTC	TCATGTCTTTGCTTTTCTTCTGG	CTTTTACATAATGGTAACTCCAGTTTA	
AS443g+	gAGATGTCTTTGCTTTTCTTC	$2	7	152904411	152904604	194	ATCTGCAAACGCAGTCAGACCTTCATC	CATTGCCTTTGCTTTTCTTCTGG	CCACTGGCAACTACAGTGACTTTTCAA	
S486G+	GCAAAGATTCCATAATATAAG	$3	15	46741100	46741285	186	GCGAAGTTGCAATGCCATCTTTC	AAAAGATCCCATAATATAAGCGG	GTTTAGTCTGCTGCTTTTTGCTGT	ccgcttatattatgggatctttt
S486G+	GCAAAGATTCCATAATATAAG	$4	10	83139568	83139750	183	GTTGGCTCACAGGACATCACTTAC	CTGTGAGTCCATAATATAAGAGG	GTTTTCCAGAATGGCTGTATCATTTTA	
S563g+	gAACCCAGCTGTAATCATGCA	$5	1	61020937	61021129	193	CACACAAATGTTCATTTTACACAAATG	AACTGTGGTGTAATCATGCAAGG	GCTATGCTGAGTAAAAGAAGCCAG	
AS706g+	gATATTGTAATGGGCTCTGTC	$6	20	53025863	53026065	203	TGAGAGCTTGAGTATAAGAGTGCTTAC	TTAGGGAAATGGGCTCTGTCAGG	CCATTTTACATTAGTCACGTCTAAC	
AS706g+	gATATTGTAATGGGCTCTGTC	$7	22	27938157	27938349	193	CTCTTCAGGCCTGTGCCCTCTGCAAC	CTAGAGGAATGGGCTCTGTCTGG	CCAGCCTGGGTCAGGCTATGTCCGT	
AS859G	GGATCAGCCATGGTAGATTA	$8	15	46430928	46431120	193	CAGCCACAGTTATGGTTTCTACTTGG	TGAGTTGCCATGGTAGATTATGG	ATTTTAGCAATGAGTTTTCCCTATTCCC	ccataatctaccatggcaactca
S893G	GCAGGTACCAATGGGGAAGA	$9	1	201056084	201056286	203	AAGATAAATGTTTCCTATGCAAGTTTAC	GATGATACCAATGGGGAAGAAGG	GAACAGCTCTCATTAATAATGACCATTA	
S893G	GCAGGTACCAATGGGGAAGA	$10	1	97836857	97837049	193	GCAAATATTAATCAGGCTCTGACCATG	TCACATAGCAATGGGGAAGAGGG	GTTCAAGACTTTGGAACTTAGAGAGG	

Note: CRISPRdirect (https://crispr.dbcls.jp/) was used to search for putative off-target sites of 8 gRNAs under the conditions of complete match of 12 nucleotides at the 3'-end and no more than four mismatches in the rest of 7 nucleotides in the 5'-region.

Supplementary Table S3. The number of sequence reads and the frequency of indels at each site

off-target site	HCK1T									SiHa					
	Ad-mock Lenti-N7B1P1H1			Ad-CB-Cas9n (500) Ad-Set2 (500)			Ad-CB-Cas9 (500) Ad-Set2 (500)			Ad-CB-Cas9n (500) Ad-Set2 (500)			Ad-CB-Cas9 (500) Ad-Set2 (500)		
	Total reads	Indel reads	Indel rate*	Total reads	Indel reads	Indel rate	Total reads	Indel reads	Indel rate	Total reads	Indel reads	Indel rate	Total reads	Indel reads	Indel rate
$1	105094	1064	0.01012	100558	1356	0.013485	106455	1520	0.014278	91567	1184	0.012930	84983	1256	0.014779
$2	109067	4699	0.04308	98744	5238	0.053046	110404	5564	0.050397	95570	4932	0.051606	82904	4510	0.054400
$3	97600	825	0.00845	102102	914	0.008952	110111	3467	0.031486	93400	835	0.008940	84297	36749	0.435947
$4	63039	1291	0.02048	66513	1325	0.019921	60432	1236	0.020453	53077	1076	0.020272	51083	1037	0.020300
$5	47424	962	0.02029	58287	1820	0.031225	53042	2050	0.038649	49632	1577	0.031774	22395	842	0.037598
$6	18479	629	0.03404	7309	298	0.040772	17908	883	0.049308	20438	704	0.034446	20401	816	0.039998
$7	48137	3712	0.07711	57170	5290	0.092531	65692	4009	0.061027	37904	2290	0.060416	35587	2887	0.081125
$8	101848	742	0.00729	91842	743	0.008090	105943	798	0.007532	86018	663	0.007708	87099	673	0.007727
$9	49726	467	0.00939	67211	793	0.011799	76036	1038	0.013651	55811	597	0.010697	50108	877	0.017502
$10	70405	3969	0.05637	68045	4221	0.062032	73969	4744	0.064135	63278	3173	0.050144	67002	2311	0.034492

*Indel rate was calculated by dividing the number of indel reads by that of total reads.

Supplementary Table S4. SNVs in the target sequences of Set2 and SetA

	Core (B1P1H1)		Set2 (N7B1P1H1)		SetA (B1P1H1Z1)	
cleaved sites	strains	%	strains	%	strains	%
4 sites	n.a.	n.a.	579	89.8	622	96.0
>3 sites	633	97.7	645	99.5	646	99.7
>2 sites	646	99.7	648	100	648	100
<=1 site	648	100	648	100	648	100

Supplementary Table S5. List of gRNA expressing rerovirus vectors and oligonucleotides used for mutagenesis

Retrovirus vector plasmid	Forward primer (5' to 3')	Reverse primer (5' to 3')	Template plasmid	reaction
pSI-CMSCVpuro-U6H1R-16S107gA+	AAAAGAGAACTGCAATGTTTCgttttagagctagaaatagca	GAAACATTGCAGTTCTCTTTTcgggaaagagtggtctcat	pSI-CMSCVpuro-U6H1R-gRNA-GFP-T2	in fusion
pSI-CMSCVpuro-U6H1R-16S485G	GCAAAGATTCCATAATATAAgttttagagctagaaatagca	CTTATATTATGGAATCTTTGcgggaaagagtggtctcat	pSI-CMSCVpuro-U6H1R-gRNA-GFP-T2	in fusion
pSI-CMSCVpuro-U6H1R-16S486G+	CAAAGATTCCATAATATAAGgttttagagctagaaatagca	CTTATATTATGGAATCTTTGcgggaaagagtggtctcat	pSI-CMSCVpuro-U6H1R-gRNA-GFP-T2	in fusion
pSI-CMSCVpuro-U6H1R-16S490G	ATTCCATAATATAAGGGGTgttttagagctagaaatagca	ACCCCTTATATTATGGAATcgggaaagagtggtctcat	pSI-CMSCVpuro-U6H1R-gRNA-GFP-T2	in fusion
pSI-CMSCVpuro-U6H1R-16S492g	CCATAATATAAGGGGTCGGgttttagagctagaaatagca	CCGACCCCTTATATTATGGcgggaaagagtggtctcat	pSI-CMSCVpuro-U6H1R-gRNA-GFP-T2	in fusion
pSI-CMSCVpuro-U6H1R-16S498g	ATATAAGGGGTCGGTGGACgttttagagctagaaatagca	GTCCACCGACCCCTTATATcgggaaagagtggtctcat	pSI-CMSCVpuro-U6H1R-gRNA-GFP-T2	in fusion
pSI-CMSCVpuro-U6H1R-16S563g	ACCCAGCTGTAATCATGCAgttttagagctagaaatagca	TGCATGATTACAGCTGGGTcgggaaagagtggtctcat	pSI-CMSCVpuro-U6H1R-gRNA-GFP-T2	in fusion
pSI-CMSCVpuro-U6H1R-16S893G	CAGGTACCAATGGGGAAGAgttttagagctagaaatagca	TCTTCCCCATTGGTACCTGcgggaaagagtggtctcat	pSI-CMSCVpuro-U6H1R-gRNA-GFP-T2	in fusion
pSI-CMSCVpuro-U6H1R-18S689g+	GAGCAATTAAGCGACTCAGgttttagagctagaaatagca	CTGAGTCGCTTAATTGCTCcgggaaagagtggtctcat	pSI-CMSCVpuro-U6H1R-gRNA-GFP-T2	in fusion
pSI-CMSCVbsd-U6H1R-16AS57G	(CTTTTATACTAACCGGTTTgttttagagctagaaatagca)	(AAACCGGTTAGTATAAAAGcgggaaagagtggtctcata)	pENTR221-U6H1R-16AS57G	LR
pSI-CMSCVbsd-U6H1R-16AS63G+	ATGTCTGCTTTTATACTAACgttttagagctagaaatagca	GTTAGTATAAAAGCAGACATcgggaaagagtggtctcat	pSI-CMSCVbsd-U6H1R-gRNA-HPV16_AS57G	in fusion
pSI-CMSCVbsd-U6H1R-16AS443g+	AGATGTCTTTGCTTTTCTTCgttttagagctagaaatagca	GAAGAAAAGCAAAGACATCTcgggaaagagtggtctcat	pSI-CMSCVbsd-U6H1R-gRNA-HPV16_AS57G	in fusion
pSI-CMSCVbsd-U6H1R-16AS507G	CAACAAGACATACATCGACgttttagagctagaaatagca	GTCGATGTATGTCTTGTTGcgggaaagagtggtctcat	pSI-CMSCVbsd-U6H1R-gRNA-HPV16_AS57G	in fusion
pSI-CMSCVbsd-U6H1R-16AS706g	TATTGTAATGGGCTCTGTCgttttagagctagaaatagca	GACAGAGCCCATTACAATAcggaaagagtggtctcat	pSI-CMSCVbsd-U6H1R-gRNA-HPV16_AS57G	in fusion
pSI-CMSCVbsd-U6H1R-16AS706g+	TATTGTAATGGGCTCTGTCgttttagagctagaaatagca	GACAGAGCCCATTACAATATcgggaaagagtggtctcat	pSI-CMSCVbsd-U6H1R-gRNA-HPV16_AS57G	in fusion
pSI-CMSCVbsd-U6H1R-16AS717G+	AAAAGGTTACAATATTGTAAgttttagagctagaaatagca	TTACAATATTGTAACCTTTTcgggaaagagtggtctcat	pSI-CMSCVbsd-U6H1R-gRNA-HPV16_AS57G	in fusion
pSI-CMSCVbsd-U6H1R-16AS859G	GATCAGCCATGGTAGATTAgttttagagctagaaatagca	TAATCTACCATGGCTGATCcgggaaagagtggtctcat	pSI-CMSCVbsd-U6H1R-gRNA-HPV16_AS57G	in fusion

Supplementary Table S6. List of lentivirus vectors expressing double-nicking gRNAs and the entry generated by multiple Gateway reactions

nickname	Lentivirus vector plasmid	Destination vector	Entry plasmid 1	Entry plamid 2	Entry plamid 3
N1	CSII-U6H1R-16S107gA-16AS57G-PGKNeo	CSII-RfA	pENTR221(L1-L4)-U6/H1R-16S107gA	pENTR221(R4r-R3r)-U6/H1R-16AS57G	pENTR221(L3-L2)-PGKNeo
N2	CSII-U6H1R-16S107g+-16AS57G-PGKNeo	CSII-RfA	pENTR221(L1-L4)-U6/H1R-16S107g+	pENTR221(R4r-R3r)-U6/H1R-16AS57G	pENTR221(L3-L2)-PGKNeo
N3	CSII-U6H1R-16S116g+-16AS57G-PGKNeo	CSII-RfA	pENTR221(L1-L4)-U6/H1R-16S116g+	pENTR221(R4r-R3r)-U6/H1R-16AS57G	pENTR221(L3-L2)-PGKNeo
N4	CSII-U6H1R-16S107g+-16AS63G+-PGKNeo	CSII-RfA	pENTR221(L1-L4)-U6/H1R-16S107g+	pENTR221(R4r-R3r)-U6/H1R-16AS63G+	pENTR221(L3-L2)-PGKNeo
N5	CSII-U6H1R-16S490G-16AS443g+-PGKNeo	CSII-RfA	pENTR221(L1-L4)-U6/H1R-16S490G	pENTR221(R4r-R3r)-U6/H1R-16AS443g+	pENTR221(L3-L2)-PGKNeo
N6	CSII-U6H1R-16S485G-16AS443g+-PGKNeo	CSII-RfA	pENTR221(L1-L4)-U6/H1R-16S485G	pENTR221(R4r-R3r)-U6/H1R-16AS443g+	pENTR221(L3-L2)-PGKNeo
B1	CSII-U6H1R-16S563G+-16AS507G-PGKBsd	CSII-RfA	pENTR221(L1-L4)-U6/H1R-16S563G+	pENTR221(R4r-R3r)-U6/H1R-16AS507G	pENTR221(L3-L2)-PGKBsd
B2	CSII-U6H1R-16S563g-16AS507G-PGKBsd	CSII-RfA	pENTR221(L1-L4)-U6/H1R-16S563g	pENTR221(R4r-R3r)-U6/H1R-16AS507G	pENTR221(L3-L2)-PGKBsd
P1	CSII-U6H1R-16S753G-16AS706g+-PGKpuro	CSII-RfA	pENTR221(L1-L4)-U6/H1R-16S753G	pENTR221(R4r-R3r)-U6/H1R-16AS706g+	pENTR221(L3-L2)-PGKpuro
P2	CSII-U6H1R-16S753G-16AS706g-PGKpuro	CSII-RfA	pENTR221(L1-L4)-U6/H1R-16S753G	pENTR221(R4r-R3r)-U6/H1R-16AS706g	pENTR221(L3-L2)-PGKpuro
P3	CSII-U6H1R-16S753G-16AS717g+-PGKpuro	CSII-RfA	pENTR221(L1-L4)-U6/H1R-16S753G	pENTR221(R4r-R3r)-U6/H1R-16AS717g+	pENTR221(L3-L2)-PGKpuro
H1	CSII-U6H1R-16S893G-16AS859G-PGKHyg	CSII-RfA	pENTR221(L1-L4)-U6/H1R-16S893G	pENTR221(R4r-R3r)-U6/H1R-16AS859G	pENTR221(L3-L2)-PGKHyg
H2	CSII-U6H1R-16S884G-16AS840g+-PGKHyg	CSII-RfA	pENTR221(L1-L4)-U6/H1R-16S884G	pENTR221(R4r-R3r)-U6/H1R-16AS840g+	pENTR221(L3-L2)-PGKHyg
Z1	CSII-U6H1R-16S1471g+-16AS1438g+-PGKzeo	CSII-RfA	pENTR221(L1-L4)-U6/H1R-16S1471g+	pENTR221(R4r-R3r)-U6/H1R-16AS1438g+	pENTR221(L3-L2)-PGKzeo
Z2	CSII-U6H1R-16S1583G-16AS1543G-PGKzeo	CSII-RfA	pENTR221(L1-L4)-U6/H1R-16S1583G	pENTR221(R4r-R3r)-U6/H1R-16AS1543G	pENTR221(L3-L2)-PGKzeo

Supplementary Table S7. List of the entry gRNA expressing cassette plasmids and oligonucleotides used for mutagenesis or cloning

Retrovirus vector plasmid	Forward primer (5' to 3')	Reverse primer (5' to 3')	Template plasmid	reaction
pENTR221(R4r-R3r)-U6H1R-16AS57G	CTTTTATACTAACCGGTTTgttttagagctagaaatagca	AAACCGGTTAGTATAAAAGcgggaaagagtggtctcat	pENTR221(R4r-R3r)-U6/H1R-gRNA-GFP-T1	in-fusion
pENTR221(R4r-R3r)-U6/H1R-16AS63G+	ATGTCTGCTTTTATACTAACgttttagagctagaaatagca	GTTAGTATAAAAGCAGACATcgggaaagagtggtctcat	pENTR221(R4r-R3r)-U6/H1R-gRNA-GFP-T1	in-fusion
pENTR221(L1-L4)-U6/H1R-16S107g+	AAAGAGAACTGCAATGTTTCgttttagagctagaaatagca	GAAACATTGCAGTTCTCTTTcgggaaagagtggtctcat	pENTR221(L1-L4)-U6/H1R-gRNA-GFP-T2	in-fusion
pENTR221(L1-L4)-U6/H1R-16S107gA+	AAAAGAGAACTGCAATGTTTCgttttagagctagaaatagca	GAAACATTGCAGTTCTCTTTTcgggaaagagtggtctcat	pENTR221(L1-L4)-U6/H1R-gRNA-GFP-T2	in-fusion
pENTR221(L1-L4)-U6/H1R-16S116g+	TGCAATGTTTCAGGACCCACgttttagagctagaaatagca	GTGGGTCCTGAAACATTGCAcgggaaagagtggtctcat	pENTR221(L1-L4)-U6/H1R-gRNA-GFP-T2	in-fusion
pENTR221(R4r-R3r)-U6/H1R-16AS507G	CAACAAGACATACATCGACgttttagagctagaaatagca	GTCGATGTATGTCTTGTTGcgggaaagagtggtctcat	pENTR221(R4r-R3r)-U6/H1R-gRNA-GFP-T1	in-fusion
pENTR221(L1-L4)-U6/H1R-16S485G	GCAAAGATTCCATAATATAAgttttagagctagaaatagca	CTTATATTATGGAATCTTTGcgggaaagagtggtctcat	pENTR221(L1-L4)-U6/H1R-gRNA-GFP-T2	in-fusion
pENTR221(L1-L4)-U6/H1R-16S490G	ATTCCATAATATAAGGGGTgttttagagctagaaatagca	ACCCCTTATATTATGGAATcgggaaagagtggtctcat	pENTR221(L1-L4)-U6/H1R-gRNA-GFP-T2	in-fusion
pENTR221(L1-L4)-U6/H1R-16S563g	ACCCAGCTGTAATCATGCAgttttagagctagaaatagca	TGCATGATTACAGCTGGGTcgggaaagagtggtctcat	pENTR221(L1-L4)-U6/H1R-gRNA-GFP-T2	in-fusion
pENTR221(R4r-R3r)-U6/H1R-16AS57G	CTTTTATACTAACCGGTTTgttttagagctagaaatagca	AAACCGGTTAGTATAAAAGcgggaaagagtggtctcat	pENTR221(R4r-R3r)-U6/H1R-gRNA-GFP-T1	in-fusion
pENTR221(L1-L4)-U6/H1R-16S753G	CAAGTGTGACTCTACGCTTgttttagagctagaaatagca	AAGCGTAGAGTCACACTTGcgggaaagagtggtctcat	pENTR221(L1-L4)-U6/H1R-gRNA-GFP-T2	in-fusion
pENTR221(L1-L4)-U6/H1R-16S563G+	ACCCAGCTGTAATCATGCAgttttagagctagaaatagca	TGCATGATTACAGCTGGGTTcgggaaagagtggtctcat	pENTR221(L1-L4)-U6/H1R-gRNA-GFP-T2	in-fusion
pENTR221(R4r-R3r)-U6/H1R-16AS706g+	TATTGTAATGGGCTCTGTCgttttagagctagaaatagca	GACAGAGCCCATTACAATATcgggaaagagtggtctcat	pENTR221(R4r-R3r)-U6/H1R-gRNA-GFP-T1	in-fusion
pENTR221(R4r-R3r)-U6/H1R-16AS706g	TATTGTAATGGGCTCTGTCgttttagagctagaaatagca	GACAGAGCCCATTACAATAcgggaaagagtggtctcat	pENTR221(R4r-R3r)-U6/H1R-gRNA-GFP-T1	in-fusion
pENTR221(R4r-R3r)-U6/H1R-16AS717g+	AAAAGGTTACAATATTGTAAgttttagagctagaaatagca	TTACAATATTGTAACCTTTTcgggaaagagtggtctcat	pENTR221(R4r-R3r)-U6/H1R-gRNA-GFP-T1	in-fusion
pENTR221(L1-L4)-U6/H1R-16S884G	GCTGATCCTGCAGGTACCAAgttttagagctagaaatagca	TTGGTACCTGCAGGATCAGcgggaaagagtggtctcat	pENTR221(L1-L4)-U6/H1R-gRNA-GFP-T2	in-fusion
pENTR221(R4r-R3r)-U6/H1R-16AS840g+	ATGGTTTCTGAGAACAGATGgttttagagctagaaatagca	CATCTGTTCTCAGAAACCATcgggaaagagtggtctcat	pENTR221(R4r-R3r)-U6/H1R-gRNA-GFP-T1	in-fusion
pENTR221(R4r-R3r)-U6/H1R-16AS859G	GATCAGCCATGGTAGATTAgttttagagctagaaatagca	TAATCTACCATGGCTGATCcgggaaagagtggtctcat	pENTR221(R4r-R3r)-U6/H1R-gRNA-GFP-T1	in-fusion
pENTR221(L1-L4)-U6/H1R-16S893G	CAGGTACCAATGGGGAAGAgttttagagctagaaatagca	TCTTCCCCATTGGTACCTGcgggaaagagtggtctcat	pENTR221(L1-L4)-U6/H1R-gRNA-GFP-T2	in-fusion
pENTR221(R4r-R3r)-U6/H1R-16AS1438g+	ACATTTAAAATATTTGTAAGgttttagagctagaaatagca	CTTACAAATATTTTAAATGTcgggaaagagtggtctcat	pENTR221(R4r-R3r)-U6/H1R-gRNA-GFP-T1	in-fusion
pENTR221(R4r-R3r)-U6/H1R-16AS1543G	TTGATTTATTACTTTTAAAgttttagagctagaaatagca	TTTAAAAGTAATAAATCAAcgggaaagagtggtctcat	pENTR221(R4r-R3r)-U6/H1R-gRNA-GFP-T1	in-fusion
pENTR221(L1-L4)-U6/H1R-16S1471g+	ACTAAAAACTAGTAATGCAAgttttagagctagaaatagca	TTGCATTACTAGTTTTTAGTcgggaaagagtggtctcat	pENTR221(L1-L4)-U6/H1R-gRNA-GFP-T2	in-fusion
pENTR221(R4r-R3r)-U6/H1R-16AS443g+	AGATGTCTTTGCTTTTCTTCgttttagagctagaaatagca	GAAGAAAAGCAAAGACATCTcgggaaagagtggtctcat	pENTR221(R4r-R3r)-U6/H1R-gRNA-GFP-T1	in-fusion
pENTR221(L1-L4)-U6/H1R-16S1583G	ATTGGTGTATTGCTGCATTgttttagagctagaaatagca	AATGCAGCAATACACCAATcgggaaagagtggtctcat	pENTR221(L1-L4)-U6/H1R-gRNA-GFP-T2	in-fusion
pENTR221(L3-L2)-PGKpuro	GGGGACAACTTTGTATAATAAAGTTGagatctaattctaccgggtag	GGGGACCACTTTGTACAAGAAAGCTGGGTAttaGGCACCGGGCTTGCGGGTC	pCMSCVpuro	BP
pENTR221(L3-L2)-PGKNeo	GGGGACAACTTTGTATAATAAAGTTGagatctaattctaccgggtag	GGGGACCACTTTGTACAAGAAAGCTGGGTAttaGAAGAACTCGTCAAGAAGGCG	pCMSCVneo	BP
pENTR221(L3-L2)-PGKHyg	GGGGACAACTTTGTATAATAAAGTTGagatctaattctaccgggtag	GGGGACCACTTTGTACAAGAAAGCTGGGTAttaTTCCTTTGCCCTCGGACGAGTG	pCMSCVhyg	BP
pENTR221(L3-L2)-PGKBsd	GGGGACAACTTTGTATAATAAAGTTGagatctaattctaccgggtag	GGGGACCACTTTGTACAAGAAAGCTGGGTAttaGCCCTCCCACACATAACCAGAG	pCMSCVbsd	BP
pENTR221(L3-L2)-PGKzeo	GGGGACAACTTTGTATAATAAAGTTGagatctaattctaccgggtag	AGAAAGCTGGGttaGTCCTGCTCCTCGGCCACGAAGT and GGGGACCACTTTGTACAAGAAAGCTGGGT	pCMSCVzeo	BP

Supplementary Table S8. List of oligonucleotides used to construct multiplex eight gRNA expression units targeting HPV16 genome.

Set	Part	Unit	Name	Nucleotide sequence
**HPV16 Set 2 (N7B1P1H1)**	**Head 8 oligos (N7B1)**	(h unit 1)	HP16-2A443ga-h1F	5′-AGGACGAGGATCC gAGATGTCTTTGCTTTTCTTC-3′
			HP16-2A443ga-h1R	3'-GCTCCTAGG cTCTACAGAAACGAAAAGAAG CAAA-5'
		(h unit 2)	HP16-2S486aG-h2F	5′-GGACGAGGATCC GCAAAGATTCCATAATATAAG G-3′
			HP16-2S486aG-h2R	3'-CTCCTAGG CGTTTCTAAGGTATTATATTC CAAAA-5'
		(h unit 3)	HP16-3A507G-h3F	5′-GACGAGGATCC GCAACAAGACATACATCGAC GT-3′
			HP16-3A507G-h3R	3'-TCCTAGG CGTTGTTCTGTATGTAGCTG CAAAAT-5'
		(h unit 4)	HP16-3S563ga-h4F	5′-ACGAGGATCC gAACCCAGCTGTAATCATGCA GTT-3′
			HP16-3S563ga-h4R	3'-CCTAGG cTTGGGTCGACATTAGTACGT CAAAATC-5'
	**Middle 8 oligos (P1H1)**	(m unit 1)	HP16-4A706ga-m1F	5′-AGGACGAGGATCC gATATTGTAATGGGCTCTGTC-3′
			HP16-4A706ga-m1R	3'-GCTCCTAGG cTATAACATTACCCGAGACAG CAAA-5'
		(m unit 2)	HP16-4S753G-m2F	5′-GGACGAGGATCC GCAAGTGTGACTCTACGCTT G-3′
			HP16-4S753G-m2R	3'-CTCCTAGG CGTTCACACTGAGATGCGAA CAAAA-5'
		(m unit 3)	HP16-6A859G-m3F	5′-GACGAGGATCC GGATCAGCCATGGTAGATTA GT-3′
			HP16-6A859G-m3R	3'-TCCTAGG CCTAGTCGGTACCATCTAAT CAAAAT-5'
		(m unit 4)	HP16-6S893G-m4F	5′-ACGAGGATCC GCAGGTACCAATGGGGAAGA GTT-3′
			HP16-6S893G-m4R	3'-CCTAGG CGTCCATGGTTACCCCTTCT CAAAATC-5'
**HPV16 Set A (B1P1H1Z1)**	**Head 8 oligos (B1P1)**	(h unit 1)	HP16-3A507G-h1F	5′-AGGACGAGGATCC GCAACAAGACATACATCGAC -3′
			HP16-3A507G-h1R	3'-GCTCCTAGG CGTTGTTCTGTATGTAGCTG CAAA-5'
		(h unit 2)	HP16-3S563ga-h2F	5′-GGACGAGGATCC gAACCCAGCTGTAATCATGCA G-3′
			HP16-3S563ga-h2R	3'-CTCCTAGG cTTGGGTCGACATTAGTACGT CAAAA-5'
		(h unit 3)	HP16-P3A717Ga-h3F	5′-GACGAGGATCC GAAAAGGTTACAATATTGTAA GT-3′
			HP16-P3A717Ga-h3R	3'-TCCTAGG CTTTTCCAATGTTATAACATT CAAAAT-5'
		(h unit 4)	HP16-P3S753G-h4F	5′-ACGAGGATCC GCAAGTGTGACTCTACGCTT GTT-3′
			HP16-P3S753G-h4R	3'-CCTAGG CGTTCACACTGAGATGCGAA CAAAATC-5'
	**Middle 8 oligos (H1Z1)**	(m unit 1)	HP16-6A859G-m1F	5′-AGGACGAGGATCC GGATCAGCCATGGTAGATTA -3′
			HP16-6A859G-m1R	3'-GCTCCTAGG CCTAGTCGGTACCATCTAAT CAAA-5'
		(m unit 2)	HP16-6S893G-m2F	5′-GGACGAGGATCC GCAGGTACCAATGGGGAAGA G-3
			HP16-6S893G-m2R	3'-CTCCTAGG CGTCCATGGTTACCCCTTCT CAAAA-5'
		(m unit 3)	HP16-Z1A1438ga-m3F	5′-GACGAGGATCC gACATTTAAAATATTTGTAAG GT-3′
			HP16-Z1A1438ga-m3R	3'-TCCTAGG cTGTAAATTTTATAAACATTC CAAA AT-5'
		(m unit 4)	HP16-Z1S1471ga-m4F	5′-ACGAGGATCC gACTAAAAACTAGTAATGCAA GTT-3′
			HP16-Z1S1471ga-m4R	3'-CCTAGG cTGATTTTTGATCATTACGTT CAAA ATC-5'
